# Chelating the valley of death: Deferoxamine’s path from bench to wound clinic

**DOI:** 10.3389/fmed.2023.1015711

**Published:** 2023-02-15

**Authors:** Jennifer B. Parker, Michelle F. Griffin, Mauricio A. Downer, Deena Akras, Charlotte E. Berry, Asha C. Cotterell, Geoffrey C. Gurtner, Michael T. Longaker, Derrick C. Wan

**Affiliations:** ^1^Division of Plastic and Reconstructive Surgery, Department of Surgery, Stanford University School of Medicine, Stanford, CA, United States; ^2^Institute for Stem Cell Biology and Regenerative Medicine, Stanford University School of Medicine, Stanford, CA, United States; ^3^Department of Surgery, University of Arizona College of Medicine, Tucson, AZ, United States

**Keywords:** iron chelation, skin radiation, reactive oxygen species, wound healing, irradiation, deferoxamine

## Abstract

There is undisputable benefit in translating basic science research concretely into clinical practice, and yet, the vast majority of therapies and treatments fail to achieve approval. The rift between basic research and approved treatment continues to grow, and in cases where a drug is granted approval, the average time from initiation of human trials to regulatory marketing authorization spans almost a decade. Albeit with these hurdles, recent research with deferoxamine (DFO) bodes significant promise as a potential treatment for chronic, radiation-induced soft tissue injury. DFO was originally approved by the Food and Drug Administration (FDA) in 1968 for the treatment of iron overload. However, investigators more recently have posited that its angiogenic and antioxidant properties could be beneficial in treating the hypovascular and reactive-oxygen species-rich tissues seen in chronic wounds and radiation-induced fibrosis (RIF). Small animal experiments of various chronic wound and RIF models confirmed that treatment with DFO improved blood flow and collagen ultrastructure. With a well-established safety profile, and now a strong foundation of basic scientific research that supports its potential use in chronic wounds and RIF, we believe that the next steps required for DFO to achieve FDA marketing approval will include large animal studies and, if those prove successful, human clinical trials. Though these milestones remain, the extensive research thus far leaves hope for DFO to bridge the gap between bench and wound clinic in the near future.

## Introduction

Receiving approval from the Food and Drug Administration (FDA) remains a significant hurdle for prospective drugs discovered in the laboratory to reach a patient. According to the National Institutes of Health, upwards of 90% of research projects fail, and almost 95% of drugs that successfully reach human clinical trial phases do not achieve approval (1). Described by many as the “valley of death,” the rift between basic research and approved treatment continues to grow (1). Multiple reasons are associated with the increasing divide, including unreproducible data, poor preclinical models, statistical errors, low incentives within academic settings, and the governmental funding mechanisms involved. In cases where the research itself is sound, and the drug does reach human clinical trial phases, the mean time from initiation of the first-in-human clinical studies to regulatory marketing authorization is 8 years (2).

As an example of a drug that bodes promise in bridging the gap between the bench and bedside, we describe the steps researchers have taken to achieve FDA approval of deferoxamine (DFO) for the treatment of chronic, radiation-induced soft tissue injury. In this perspective, we begin with a brief outline of the steps taken to achieve FDA marketing approval for DFO in the treatment of iron overload back in the 1960s. We then proceed with an overview of chronic wounds and radiation induced fibrosis (RIF), and describe DFO’s possible mechanism of action for the purposes of treating chronic wounds and RIF. Following this, we summarize small animal studies investigating DFO as a potential therapy for these conditions, and close with a discussion of what we believe are the anticipated next steps required to achieve FDA marketing approval of DFO for the treatment of chronic wounds and radiation-induced soft tissue injury.

## History on deferoxamine as an iron chelator

Deferoxamine was first discovered coincidentally through research conducted by Ciba, Basle, and the Swiss Federal Institute of Technology in Zurich (3). They originally aimed to discover iron-containing antibiotics known as ferrimycines (4). In the process, they also found iron-containing antibiotic antagonists known as ferrioxamines (4, 5). Ciba dropped the project shortly after it began as bacteria quickly developed resistance against ferrimycine; instead, the team focused on the ferrioxamine impurities and their potential therapeutic uses (3).

Notably, ferrioxamines contain iron, and the group hypothesized that these compounds could be used as iron supplements for patients suffering from iron-deficiency anemia (6). To the investigators’ surprise, however, it was determined that ferrioxamine is mainly excreted and little iron is displaced from ferrioxamine molecules when the drug is administered. Given the molecule’s high affinity to iron, the team changed course for a third time, now positing that an iron-free version of the molecule would be capable of binding to excess iron thereby removing it from the body. This iron free preparation, known as DFO, was first produced in December 1960 (3).

Animal experiments followed in rabbits and dogs (3, 6, 7). Demonstrating that DFO increased iron levels within the urine and that it had a safe toxicity profile, the drug was cleared for human clinical trial. After tolerability tests performed in human volunteers, the first hemochromatosis patient was treated in 1961 with promising results (6). Trials with additional patients were conducted shortly thereafter, and DFO was officially registered in Switzerland and brought to market only 2 years later in 1963 (3). The FDA approved DFO for use as an iron chelator in 1968 (8).

Since then, DFO remains approved for the treatment of iron overload in both acute and chronic settings, and has been used off-label in the treatment of aluminum toxicity in chronic kidney disease (CKD) patients (9). It has been on the World Health Organization’s List of Essential Medicines since 1979 (10). Subsequently to its use in iron overload, DFO has been explored in a number of animal models in the contexts of wound healing and RIF.

## Chronic wounds

Normal wound healing occurs in a series of stages. After injury, bleeding is controlled *via* the coagulation cascade (11). During the first week, inflammatory mediators including tumor necrosis factor-alpha (TNF-α) and transforming growth factor beta (TGF-ß) accumulate in the wound, and attract neutrophils and monocytes (12, 13). These white blood cells help remove debris and bacteria from the wound bed, and stimulate the proliferative phase of wound healing. At this point, capillaries reform to repair damage to vasculature, and fibroblasts deposit a provisional matrix and initiate wound contracture (11). Eventually, during the maturation and remodeling stage of wound healing, the temporary matrix is replaced by more organized, structured scar tissue, which completes the wound healing process. Chronic wounds fail to complete the stages of wound healing outlined. As they are often incompletely healed and open, these wounds typically lead to unresolved tissue damage, and eventual tissue necrosis (14).

Though the etiology is variable, multiple factors have been identified that drive incomplete wound healing. Tissue ischemia can reduce oxygenation and nutrient circulation, which are both necessary for the survival of cells within the wound bed. Multiple clinical factors can result in ischemia, including the location of the wound and vascular dysfunction. The lower extremities are the most common sites for chronic wounds given decreased circulation in this area relative to the rest of the body (15). Further, elderly patients are often disproportionately affected by chronic wounds as they often have increased risk of comorbid conditions that decrease wound repair, such as a higher risk of underlying ischemia, increased levels of reactive oxygen species, and cellular senescence (14, 16). Another factor that can further exacerbate underhealing in wounds is bacterial colonization (17). Though bacterial colonization occurs within 48 h of the initial injury, lack of wound closure can lead to persistent infections. During the body’s attempt to fight these infections, immune cells such as neutrophils release proteases that also damage local tissue and further lead to underhealing in wounds (17).

Chronic wounds fall under a number of different classes. Pressure injuries, or pressure ulcers, are caused by prolonged tissue compression (18). Compression of overlying skin, particularly in the situation above a bony prominence such as the sacrum, results in tissue ischemia and eventual necrosis. These wounds are most commonly seen in insensate or immobilized patients. Diabetic ulcers arise as a result of diabetic complications (19). Increased risk of peripheral artery or vascular disease in tandem with peripheral neuropathy leads to reduced healing and increased risk of tissue trauma given that patients may not be aware of injuries in their lower extremities. Vascular ulcers are another common cause of chronic wounds. Venous ulcers arise from diminished venous return, while arterial ulcers are the result of diminished blood flow (20). Finally, impaired wound healing also occurs in irradiated tissue (21). Acutely, radiation directly compromises healing due to damage radiation causes to healthy tissue. Further, in the long term, radiation can lead to extensive fibrosis in the irradiated tissue, known as RIF, and will be elaborated on in the following section.

## Radiation induced fibrosis

Broadly, radiation therapy is a key component of cancer therapy, with an estimated 60% of cancer patients receiving radiation therapy at some point during their treatment course (22). Unfortunately, though radiation therapy often improves survival, significant complications are associated with the treatment. These include erythema, desquamation, ulceration, and edema in an acute setting (23). RIF consists of the complications seen long-term secondary to radiation therapy, usually developing 4–12 months post treatment, and persisting many years after (24, 25).

Soft tissue injury following radiation parallels the mechanism seen in wound healing, with an acute inflammatory phase post radiation treatment, followed by fibroblast recruitment, and eventual matrix deposition (26). The initial ionizing radiation that causes direct damage to DNA and production of ROS results in cell damage, triggering an immune response (27). Radiation also creates added local injury *via* ischemia and thrombosis, which leads to the release of cytokines including TNF-α, interleukin (IL)-1, and IL-6 (28). The added cytokines recruit monocytes and lymphocytes. These cells secrete their own factors such as platelet-derived growth factor, which in turn attract fibroblasts to the area of injury (29). Fibroblasts deposit excess collagen, thickening the irradiated tissue while decreasing its vascularity (24, 30). These proinflammatory and profibrotic signals can remain upregulated many years after radiation therapy. RIF is considered a significant surgical complication; for example, upwards of 20% of all breast cancer patients are affected and experience increased risk of complications such as infection, capsular contracture, and surgical revision post reconstructive surgery relative to patients receiving breast reconstruction without a history of radiation therapy (31, 32).

As alluded to, wound healing is often impaired in irradiated skin due to the cellular injury, microvascular damage, and a persistent, pro-inflammatory environment that results. These changes to the wound microenvironment dysregulate the normal stages of wound healing, and commonly lead to compromised wound healing (21). For example, fibroblasts, which play a key role in the deposition of extracellular matrix within the wound site, display disorganized collagen deposition in irradiated tissue (26, 30). This is a crucial component of the proliferative and remodeling phases of wound healing, and disorganized matrix deposition affects the integrity of the wound bed. Further, keratinocytes are crucial for skin epithelialization, and keratinocytes in RIF skin often show higher levels of matrix metalloproteinase activity, which can negatively impact wound healing by delaying re-epithelialization and inhibiting wound closure (33).

Unfortunately, with both chronic wounds and RIF, the long-term changes to tissue make both of these conditions very difficult to treat. However, research within the last decade suggests that DFO bodes promise as a therapeutic. In the following section, we describe DFO’s suggested mechanism of action in chronic wounds and RIF, and summarize our group’s investigations exploring DFO treatment in a number of small animal chronic wound and RIF models.

## Deferoxamine mechanisms and potential therapeutic applications in chronic wound healing and RIF

**Figure 1:**
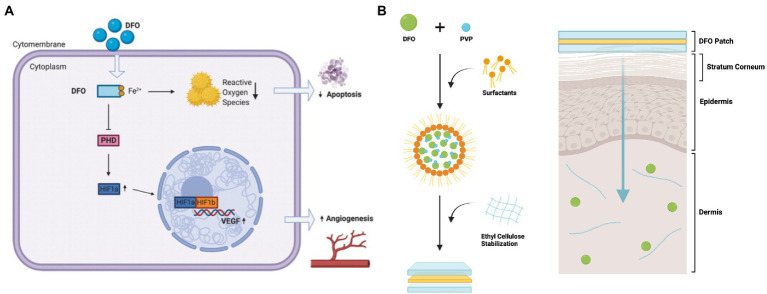
Deferoxamine (DFO)‘s potential mechanism of action and schematic of the transdermal drug delivery system for DFO. **(A)** After treatment, DFO leads to decreased cellular iron availability, which leads to increased VEGF mRNA expression and protein. With the removal of iron, a necessary cofactor for PHD, PHD is inactivated and no longer able to degrade HIF-1α. Accumulation of HIF-1α leads to activation of the HIF1 heterodimer, and triggers downstream effectors in angiogenic pathways, such as VEGF transcription and translation. DFO also reduces free radical formation and reactive oxygen stress catalyzed by iron, and thus diminishes apoptosis. Reproduced with permission from Tevlin et al. (26). **(B)** DFO is aggregated with PVP and surfactants, forming reverse micelles (RM). These RM are then stabilized in ethyl cellulose. When the patch is placed on the skin, the RM are released from the ethyl cellulose matrix and can diffuse through the hydrophobic stratum corneum. The RM then break down, releasing the DFO-PVP aggregates. The PVP then dissolves, delivering DFO to the dermis. Figure adapted from Shen et al. (34).

Deferoxamine’s angiogenic and antioxidant properties were originally posited following *in vitro* studies that showed that DFO increased vascular endothelial growth factor (VEGF) mRNA and protein production *via* the transcription factor hypoxia-inducible factor 1 (HIF-1) (Figure 1A) (35). HIF-1 and VEGF are key signaling molecules involved in the induction of angiogenesis (36). HIF-1 is a heterodimer consisting of HIF-1α and HIF-1β subunits. While HIF-1β is expressed constitutively, HIF-1α degrades during normoxia, and only becomes upregulated in hypoxic conditions (36). When tissues experience low levels of oxygen, HIF-1α accumulates, and HIF-1 heterodimerizes, activating a number of target genes including VEGF. HIF-1 degrades in the presence of an enzyme called prolyl-4-hydroxylase (PHD). This enzyme requires iron as a cofactor, and by chelating iron, DFO inactivates PHD, leading to accumulation of HIF-1α, activation of the HIF-1 heterodimer, and thus triggering the downstream angiogenic signaling pathway (37). Importantly, as DFO sequesters iron ions, DFO has also been shown to inhibit iron-catalyzed reactive oxygen stress, decreasing ROS formation and cellular apoptosis as a result (38, 39).

This suggested mechanism led to the hypothesis that DFO holds promise in improving wound healing and RIF, as both of these conditions are characterized by hypovascularity and elevated levels of ROS (27, 40). Although this drug is already FDA approved to treat iron overload, an additional New Drug Application (NDA) is required for the use of DFO in the treatment of chronic wounds and radiation-induced soft tissue injury, specifically (41). To achieve this, results from *in vivo* animal studies followed by those from human clinical trials will be required (41). Here, we will summarize key animal studies conducted by our group since 2015 that we feel will contribute critical information to an eventual NDA. Additional small animal studies exploring the use of DFO in wound healing and fibrosis are summarized in Table 1.

**Table 1:** Small animal studies exploring deferoxamine (DFO) in various wound healing and fibrosis models.

Study title	Model and methods	Results
Deferoxamine enhances neovascularization and accelerates wound healing in diabetic rats *via* the accumulation of hypoxia-inducible factor-1α [Hou et al. (42)]	Using an excisional diabetic wound model in rats, Hou et al. (42) compared deferoxamine treatment to VEGF treatment and vehicle control. Human umbilical vein endothelial cells (HUVECs) were used to study VEGF and SDF-1a expression upon DFO treatment and HIF-1α knockdown *in vitro.*	DFO treated wounds saw accelerated wound healing compared to control as illustrated by elevated granulation tissue, re-epithelization and neovascularization. *In vitro*, DFO treatment led to increased endothelial tube formation, cell proliferation and migration of HUVECs, while it did not increase VEGF expression in HIF-1α knockdown cells.
Deferoxamine-Soaked Suture Improves Angiogenesis and Repair Potential After Acute Injury of the Chicken Achilles Tendon (43)	Efird et al. (43) used an acute Achilles tendon injury model in chickens by partially transecting the right Achilles tendon. Tendons were then repaired with Vicryl suture soaked in sterile water or DFO solution, and tissue collected 2 weeks later.	DFO-soaked suture group demonstrated elevated hemoglobin content as measured by percentage of wet tissue as well as increased articular zone vessel density as compared to the control. These data suggested that the DFO-soaked suture increased angiogenesis and repair.
Co-delivery of deferoxamine and hydroxysafflor yellow A to accelerate diabetic wound healing *via* enhanced angiogenesis (44)	Rats were induced for Type 1 Diabetes Mellitus underwent excisional dorsal wounding. Wounds were treated with DFO, hydroxysafflor yellow A (HSYA), combination DFO/HSYA, or PBS daily and mice harvested after 4 weeks. Prior to this study, HYSA was previously shown to accelerate diabetic wound healing *via* improved angiogenesis and decreased inflammation.	DFO and HSYA in combination demonstrated accelerated wound closure and improved vasculature relative to other groups.
Topical Deferoxamine Alleviates Skin Injury and Normalizes Atomic Force Microscopy Patterns Following Radiation in a Murine Breast Reconstruction Model (45)	A rat model of expander-based breast reconstruction and irradiation was used. Silicon-based mini-expanders were placed in sub-musculocutaneous pockets in rats, and expanded on postoperative days 15, 18, and 21. Rats next underwent irradiation for 5 days starting on postoperative day 22. A DFO patch was then placed on top of the expanded and irradiated tissue for 10 days, after which rats were sacrificed and tissue collected. The irradiated expander group with DFO patch was compared to irradiated expander and non-irradiated expander control groups.	Topical DFO led to reduced ulceration and fibril disorganization when compared to the non-treated irradiated group. No statistical difference was observed between the DFO treatment and non-irradiated control groups.
Topical bilirubin-deferoxamine hastens excisional wound healing by modulating inflammation, oxidative stress, angiogenesis, and collagen deposition in diabetic rats (46)	Using a diabetic excisional wound model in rats, wounds were treated with a topical application of a combination bilirubin-DFO cream, and compared to vehicle control wounds.	Treatment significantly increased wound contraction. Further, treated wounds demonstrated increased angiogenesis as shown by upregulation of VEGF, HIF-1α and CD31, as well as improved collagen deposition and fibroblast proliferation. These data suggested that the bilirubin-DFO ointment led to improved diabetic wound healing.
Deferoxamine preconditioning to restore impaired HIF-1α-mediated angiogenic mechanisms in adipose-derived stem cells from STZ-induced type 1 diabetic rats (47)	Adipocyte-derived stem cells (ADSCs) were isolated from the fat pads of diabetic and non-diabetic rats. The three groups of cultured ADSCs consisted of non-diabetic control ADSCs, diabetic ADSCs, and diabetic ADSCs treated with DFO. Supernatant from the ADSCs culture was concentrated *via* ultra-filtration. Concentrated culture medium was then injected into excisional wounds of healthy rats. Wound tissue was then collected at days 3, 7, 10, and 15.	Culture medium derived from DFO pre-treated diabetic ADSCs accelerated wound closure and increased angiogenesis in comparison to culture medium derived from non-treated diabetic ADSCs. DFO pre-treated diabetic ADSC culture medium also led to increased collagen deposition and epithelialization in wounds relative to treatment with diabetic ADSC culture medium.
Deferoxamine can prevent pressure ulcers and accelerate healing in aged mice (48)	Bonham et al. (48) used a pressure ulcer model in aged mice (defined as 21 months of age or older) by placing skin from the mouse’s dorsum between two ceramic magnets for a period of 6 h to induce ischemia and forming two distinct ulcers on their back. These ulcers were injected with either PBS control or DFO. Injections began a day prior to ulcer formation, and continued every other day until wounds closed.	DFO treatment decreased pressure ulcer formation and lessened the grade of ulceration. Pressure ulcers treated with DFO also demonstrated increased CD31 expression, suggesting that DFO treatment enhanced neovascularization. Cell death *via* a TUNEL assay was also significantly decreased in the treatment group relative to control. Results suggested overall that DFO may be effective in preventing and treating pressure ulcers in elderly patients.
Pressure-driven spreadable deferoxamine-laden hydrogels for vascularized skin flaps (49)	Using an *in vivo* flap model, rats were split into a control group, a vehicle control [consisting of a beta-sheet-rich silk nanofiber (BSNF) hydrogel], and a DFO-laden BSNF hydrogel group. Ping pong-shaped flaps were used to assess the effect of DFO treatment on vascularneogenesis and necrosis. To do so, racket-shaped flaps on the dorsi of rats were incised with a scalpel, and the hydrogels were then pushed under the flap, and the flap sutured closed.	DFO-loaded BSNF hydrogel resulted in increased angiogenesis and overall flap survival relative to vehicle and control.

### Diabetic ulcers and combination product development

One of the first groups to investigate DFO as a treatment for wounds made use of a murine diabetes model (38). The study of wound healing in diabetics is of particular interest as the most common cause of non-traumatic amputation in the United States is the result of diabetic non-healing wounds (50). Importantly, HIF-1α function is compromised in diabetic patients (51). Upon administration of DFO, levels of iron-catalyzed ROS which normally interfere with HIF-1α function decreased, thus correcting HIF-1 function (38). Duscher demonstrated that their treatment, when applied to a pressure-induced ulcer model in diabetic mice, led to significantly accelerated healing relative to a non-treatment control (38).

Notably, the team chose to design a transdermal delivery modality as DFO has a short plasma half-life, and systemic treatment in diabetic patients has been associated with toxicity. Transdermal delivery is complicated by the fact that DFO has a relatively high atomic mass and is hydrophilic (52). These properties prevent the compound from penetrating passively through the stratum corneum, the lipophilic outer layer of the skin. To circumvent this, the group developed a matrix type transdermal drug delivery system (TDDS) that encapsulates DFO within a biodegradable polymer (Figure 1B) (38). In the polymer, reverse micelles enclose the DFO molecules, allowing for delivery of the drug through the hydrophobic stratum corneum. Once past this barrier, the reverse micelles break down, releasing drug into the dermis. From an FDA standpoint, the DFO TDDS is known as a combination product as it consists of a combination of a drug and device used for therapeutic purposes (53).

### Radiation induced fibrosis

Autologous fat grafting has become an increasingly popular strategy to help treat soft tissue deficiencies in the body. Unfortunately, transfer of avascular fat to a region that is fibrotic and already has depleted vasculature due to previous radiation therapy is challenging, and these fat grafts often exhibit decreased graft retention relative to grafts placed in non-irradiated patients (24, 54). Flacco investigated whether preconditioning irradiated tissue with DFO injected subcutaneously could lead to improved fat graft retention (55). Immunocompromised mice were treated after irradiation with DFO and then underwent grafting with human lipoaspirate. With DFO treatment, improved vascularity in chronic radiation injured tissue was appreciated and this facilitated greater fat graft retention. Therefore, DFO injections may be effective in reversing some of the pathologic changes in skin and soft tissue following radiation therapy, and in creating a less hostile niche for subsequent fat grafting.

Prophylactic treatment with transdermal DFO in RIF was further explored by Shen et al. (34). Making use of the TDDS DFO patch Duscher et al. developed, the team treated immunodeficient mice before and/or after radiation therapy (38). A significant decrease in ROS and apoptotic markers was seen in mice who had received prophylactic TDDS DFO. Mice in the treatment group also experienced increased skin perfusion *via* laser doppler along with decreased dermal thickness and improved collagen fiber network organization. All of these data further confirm that DFO improves tissue perfusion, and could help mitigate chronic RIF in the skin.

### Wounds in combination with radiation

Due to the characteristics of dermal RIF, wound healing can pose a significant problem for patients needing reconstruction in irradiated areas, with an estimated 35% of patients incurring major wound healing complications in irradiated areas post-operatively (56). Lintel and Abbas sought to evaluate DFO as a potential therapy for acute wounds in chronically irradiated skin (57). Following induction of dermal RIF, the team created excisional wounds within irradiated tissue. Mice were then treated with the TDDS DFO patch or a vehicle control. Non-irradiated wounds were also used as an additional comparison. Their results demonstrate that DFO accelerated wound closure relative to irradiated wound controls. DFO also improved perfusion and collagen fiber organization, and increased wound thickness and collagen density relative to irradiated wounds. These data demonstrate the therapeutic potential of TDDS DFO as a treatment to promote wound healing in patients undergoing surgery following radiotherapy.

### Treatment modality investigations

Previous studies made use of a number of different DFO administration methods, including topical and injection-based approaches. In order to evaluate the effectiveness of different treatment modalities, Lavin et al. used a similar murine RIF model as previously described and administered different treatment modalities of DFO to compare their effects on RIF (58). They showed that DFO as both a TDDS patch and an injection decreased dermal thickness and collagen content, and improved collagen fiber assembly, dermal elasticity, and skin perfusion. Notably, however, the team concluded that TDDS DFO patch treatment, particularly if administered pre and post irradiation, offered better results than DFO treatment *via* injection.

## Discussion and future directions: Path to NDA approval

The small animal studies summarized above underscore the therapeutic potential of DFO in both wound healing and RIF, and demonstrate the promise of the TDDS DFO patch as a delivery modality. In terms of next steps, larger animal studies will be critical to achieve FDA approval for the use of DFO in the treatment of these conditions. Though a previous study was conducted in a porcine model in which Weinstein et al. confirmed the beneficial effects of DFO in the context of ischemic flaps, no study evaluating transdermal and topical DFO administration routes in a large animal model has been published to date (59). Large animal studies have been used in the study of diabetic wounds and pressure ulcers, which could be applied to DFO wound healing studies (60, 61). Although no RIF models in large animals have been published to date, our group believes that scaling up the mouse RIF in a larger animal such as pig would be feasible. No small or large animal study to our knowledge has been developed for the study of chronic wounds.

If trials with a large animal model are successful, then stage 1 clinical trials testing the safety of the transdermal administration route of DFO in human volunteers can begin. With safety confirmed, investigators can then move on to treating patients in stage 2 and 3 trials. It is after these phases that results can be reviewed by the FDA and an NDA submitted (41). These studies will not only be crucial to confirm that the TDDS DFO patch is safe and effective for treatment of chronic wounds and RIF, but will also allow investigators to optimize dosage and treatment course. Further, separate studies will be necessary to evaluate the effectiveness of transdermal DFO for different conditions, such as RIF or chronic wounds. For instance, approval was granted in 2019 to conduct a randomized clinical trial to assess the effectiveness of transdermal DFO in the treatment of leg ulcers in sickle cell patients. This trial’s initial approval hints toward the prospect of future clinical trials to come that will investigate transdermal DFO use in other forms of wound healing and fibrosis (62).

One of the benefits of translational investigations with DFO lies in the fact that it has already been approved for iron overload. Further, DFO is off patent with multiple generic versions available, which facilitates its study. Referring back to the aforementioned ‘valley of death,’ the path to drug approval is expensive, time consuming, and the majority of products that start at the bench side never achieve approval. However, DFO is unique as it has been used for over a half century in human subjects and has a well-established safety profile. On top of that, a strong foundation of basic scientific research now supports its potential use in chronic wounds and RIF. Though additional milestones remain to be completed regarding DFO treatment in wound healing and fibrosis, we are hopeful to bridge that gap from bench to wound clinics in the near future.

## Data availability statement

The original contributions presented in the study are included in the article/supplementary material, further inquiries can be directed to the corresponding author.

## Author contributions

JP reviewed the literature, designed the review, contributed to the writing, and finalized the manuscript. MG contributed to the writing and review of the manuscript. MD contributed to reviewing the literature and the manuscript. DA, CB, and AC contributed to reviewing the manuscript. GG, ML, and DW oversaw the design of the review manuscript. All authors contributed to the article and approved the submitted version.

## Funding

Figure 1 was created with Biorender.com. This work was supported by the Hagey Laboratory for Pediatric Regenerative Medicine, the National Institutes of Health, and RO1 DE 027346 (to DW and ML), and the California Institute for Regenerative Medicine (EDU4-12782).

## Conflict of interest

ML and GG have equity in TauTona Group, and an incubator that produces the TDDS DFO patch.

The remaining authors declare that the research was conducted in the absence of any commercial or financial relationships that could be construed as a potential conflict of interest.

## Publisher’s note

All claims expressed in this article are solely those of the authors and do not necessarily represent those of their affiliated organizations, or those of the publisher, the editors and the reviewers. Any product that may be evaluated in this article, or claim that may be made by its manufacturer, is not guaranteed or endorsed by the publisher.
